# Behavioural and physiological expression of arousal during decision-making in laying hens^☆^

**DOI:** 10.1016/j.physbeh.2013.10.008

**Published:** 2014-01-17

**Authors:** A.C. Davies, A.N. Radford, C.J. Nicol

**Affiliations:** a Animal Welfare and Behaviour Group, School of Clinical Veterinary Science, University of Bristol, Langford House, Langford, Bristol BS40 5DU, UK; b School of Biological Sciences, University of Bristol, Woodland Road, Bristol BS8 1UG, UK

**Keywords:** Chicken, Choice, Heart-rate, Head movements, Anticipation, Motivation

## Abstract

Human studies suggest that prior emotional responses are stored within the brain as associations called somatic markers and are recalled to inform rapid decision-making. Consequently, behavioural and physiological indicators of arousal are detectable in humans when making decisions, and influence decision outcomes. Here we provide the first evidence of anticipatory arousal around the time of decision-making in non-human animals. Chickens were subjected to five experimental conditions, which varied in the number (one versus two), type (mealworms or empty bowl) and choice (same or different) of T-maze goals. As indicators of arousal, heart-rate and head movements were measured when goals were visible but not accessible; latency to reach the goal indicated motivation. We found a greater increase in heart-rate from baseline to the goal-viewing period, more head movements and shorter latencies in all conditions including mealworms compared to those with empty bowls. More head movements when two mealworm bowls were available compared to just one, and prior to occasions when hens accessed an empty bowl rather than declining to move, showed that arousal preceded and influenced decision-making. Our results provide an important foundation for investigating arousal during animal decision-making and suggest that the somatic-marker hypothesis might not only apply to humans.

## Highlights

• Anticipatory arousal was detectable around the time of decision-making in chickens.
• Increased heart-rate and head movements were measured prior to preferred goal access.
• More head movements were measured when two preferred goals were available.
• Fewer head movements were measured preceding decisions not to access a goal.
• Provides an important foundation for exploring arousal during animal decision-making.

## 1 Introduction

The theory of optimal decision-making assumes that animals have perfect knowledge of their external environment and their own internal state at any one time. In reality, it is extremely unlikely that animals have such perfect knowledge or that they are perfect processors of such knowledge. Indeed, animals frequently seem to behave in a sub-optimal manner, by making decisions that violate the principles of rationality [1–4]. However, a closer examination reveals that across a range of species, mistakes are systematic and not random [5]. Under conditions where the information available is incomplete (or overwhelming), it is not surprising that “fast-but-inaccurate” strategies are sometimes employed [6,7].

One potentially “fast-but-inaccurate” decision-making strategy, of growing interest in the human literature, concerns the role of emotion [8]. It has been suggested that when humans are faced with uncertain, complex or difficult choices, their reliance upon rational and conscious thought processes declines, and that information is channelled via short subcortical pathways, resulting in emotional reactions playing a larger role in choice behaviour [9]. Crucially, it is proposed that prior affective physiological reactions to choice options are stored within the brain as associations called somatic markers [8]. During decision-making, the somatic markers created by the relevant stimuli are summed to produce a net state that enables a rapid decision. This intriguing idea suggests that when making decisions, humans, and indeed other animals, will experience evoked emotions consciously or unconsciously associated with their past experience of each option. Behavioural and physiological signals, particularly those indicative of arousal, should therefore be detectable at around the time of decision-making and should influence the decision made.

Researchers have begun to investigate how arousal measured by skin conductance or heart-rate is associated with decision-making in humans [9–11]. Humans with deficits in emotional, but not cognitive, processing have a diminished anticipatory arousal response and make poorer decisions in gambling tasks [12]. Moreover, people who perform best during such tasks show higher anticipatory arousal preceding risky decisions that result in a net loss [10]. Behavioural and physiological expression of anticipatory arousal has also been investigated to some extent in non-human mammals and birds [13–20], although not to our knowledge during decision-making. Previously, we found that some long-term physiological reactions (e.g. blood glucose or corticosterone concentrations) to different choice options were associated with future decisions made by chickens [21,22]. However, in that work we did not assess their physiological responses at the time that decisions were made. Thus, it is not clear whether any prior emotional state influenced the arousal level of the chickens at the time of their decision.

As a first step in examining how arousal is associated with decision-making in non-human animals, we here manipulated reward outcome for chickens in a simple T-maze choice apparatus. We measured both heart-rate (HR), using a non-invasive methodology [23,24], and head movements [18] as indicators of arousal. Whilst chickens were in the T-maze start-box, we measured their baseline HR prior to any presentation of the goals. We then measured both HR and the number of head movements during a period when goals were visible but could not yet be accessed.

We investigated whether a differential arousal response occurred to conditioned stimuli indicating goals of greater (i.e. mealworms: [25]) or lesser value (i.e. an empty bowl) by examining the chickens' response to presentation of one option at a time. To check that the birds were more motivated to approach mealworms than the empty bowl, we assessed their latency to approach the reward [26]. To assess any effects of goal quantity, we provided experimental conditions where two identical options (two mealworm bowls or two empty bowls), one on each side of the T-maze, were available. The remaining test condition provided the chickens with a choice between the mealworms and the empty bowl to allow us to assess whether making a decision between goals of unequal value was associated with changes in arousal.

## 2 Materials and methods

### 2.1 Animals, housing and husbandry

Sixteen Lohmann Brown laying hens were obtained at approximately 35 weeks of age and leg-tagged for identification. They were housed on day 1 in groups of four, in four out of eight available pens (0.96 × 1.2 m, 2 m high) in the same room (home room). The home room was arranged so that opposite pens could be joined by a Perspex tunnel (1.79 × 0.24 m, 0.47 m high). Between days 1 and 20, hens were allowed to settle, being handled minimally.

Ad libitum feed (Farmgate Layers Mash, BOCM Pauls, Ipswich, Suffolk, UK) was provided via two feed troughs external to each pen. Water was available from a hanging drinker in the back corner of each pen. A nest box (0.39 × 0.38 × 0.47 m) and a round perch (0.96 m, 0.25 m high) were also provided. Wood shavings were used as bedding at a depth of 5–10 cm. During weekly cleaning, each group of four birds was switched to the opposite pen within the home room to avoid a side-housing bias. The room temperature was kept at 19–22 °C and the lighting schedule was 12 L:12 D (with lights on at 07:00).

All work was conducted under UK Home Office licence (30/2332). The hens were re-homed to small free-range holdings after the study.

### 2.2 Experimental room

The experimental room contained two pens (one on each side of the room), which were identical to those in the home room and could similarly be joined by a Perspex tunnel. The exceptions were that the feed trough openings were blocked using wooden panels and the pens did not contain nest boxes and perches. The experimental room was separated from the home room by solid wooden doors and a corridor, providing an area where hens could be tested away from the noise of conspecifics. Within the experimental room, a CCTV camera was attached to the ceiling above the test apparatus, which was connected to a computer on one side of the room. Another computer for HR monitoring was set-up on the other side of the room.

### 2.3 Habituation and training phase

Habituation (to the HR monitor and the T-maze procedure) and training (to establish an association between feed bowl and reward identity) began on day 20. Training and habituation continued until day 40.

#### 2.3.1 Harness and HR monitor

HR was monitored using a non-invasive remote telemetric unit [23]. Harnesses containing a HR monitor were made using elastane from a template designed to fit the hen without restricting movement and were fastened using press studs. The harness contained a padded integrated pocket to provide protection for the ECG cables and monitor without providing discomfort to the hen. The pocket containing the monitor was positioned over the hen's back and the ECG cables were threaded through the harness and attached to the monitor. Self-adhesive electrode sensors (Ambu Blue sensor M-00-S) were attached to pre-cleansed skin either side of the keel bone at the start of each test day.

Twelve of the 16 hens had previously experienced wearing the HR monitor and harness for an unrelated study. Additional habituation was given to the hens that had no such prior experience. Specific criteria (that hens were able to walk and behave normally in their home environment, without moving backwards or stopping excessively) had to be satisfied before individuals progressed to each next stage of HR habituation. Initially, the length of time wearing the harness alone was increased from 1 to 3 h in 30 minute increments. The ECG cables were then added to the harness and finally the monitor (weighing approx. 100 g) was added. The final few sessions of HR habituation were carried out whilst hens were trained in the experimental room, to ensure that they were able to perform the test wearing the monitor and harness. It took between 2 and 5 min to fit the hen with the heart-rate monitor and harness at the start of each session. In total, each individual wore the harness for approximately 18 h during habituation.

#### 2.3.2 Feed bowls

Hens were trained to discriminate between two different feed bowls: one, a black-and-white spotty bowl (internal diameter: 118 mm, 37 mm deep) (containing mealworms); the other, an empty beige bowl (internal diameter: 114 mm, 46 mm deep) (containing no reward). To reduce initial fear of novel stimuli, bowls were first presented to whole groups for a 1-hour period of familiarisation. After approximately three such sessions, all birds approached the bowls without signs of fearful behaviour (i.e. they approached the bowl quickly without stopping or hesitating). Training individuals to discriminate between the bowls continued during T-maze habituation. Birds were deemed able to discriminate when they consistently approached the mealworm bowl first (on at least nine out of 10 test sessions).

#### 2.3.3 T-maze test procedure

**Fig. 1:**
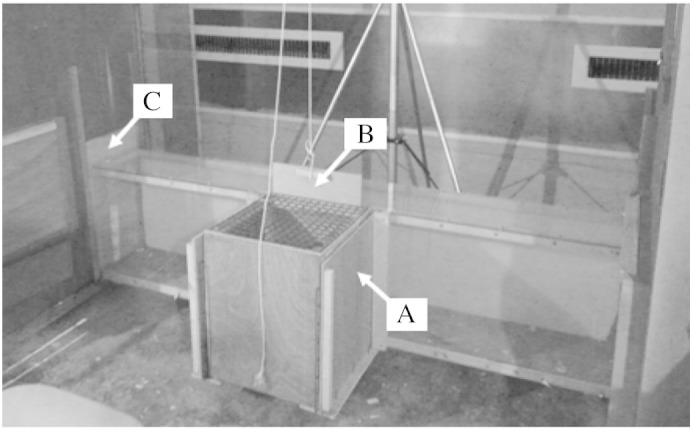
T-maze test apparatus consisting of a Perspex tunnel and attached wooden start-box. The tunnel connects the two pens in the experimental room. A indicates the wooden side-doors of the start-box which were removed to reveal wire mesh, through which the goal(s) could be viewed. B indicates the tunnel-door, which was raised using a pulley mechanism to allow access to the tunnel. C marks the pen-door (shown as closed) which was placed in the pen entrance once the hen entered the pen, to prevent her from re-entering the tunnel.

A T-maze was used to measure the behaviour of hens during each experimental condition (Fig. 1). Initial familiarisation was carried out in home groups by joining opposite home pens using the interconnecting tunnel. Two 2-hour sessions were sufficient to ensure that all hens in each group had walked through the tunnel without stopping or behaving hesitantly. Habituation to the T-maze in the experimental room was then conducted with individual birds. Initially, only the tunnel was used until individuals walked through without stopping. A start-box (0.38 × 0.39 m, 0.47 m high) was then attached to the tunnel to form the T-maze. A tunnel door suspended by a pulley mechanism at the tunnel entrance was then added, preventing hens entering the tunnel for a short period and allowing assessment of their behaviour and HR whilst they were stationary and able to view the conditioned stimuli available. To achieve this, we initially confined hens in the start-box for up to 30 s, until they showed no escape attempts or excessive vocalisations.

**Fig. 2:**
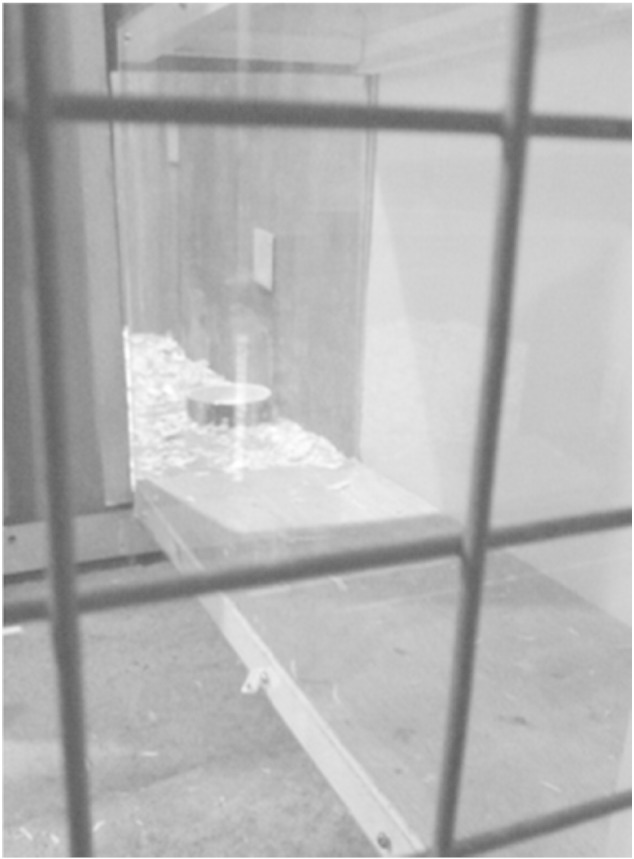
The spotty (mealworm) bowl as viewed from the start-box when the wooden side-doors were removed. The diagonal distance from the start-box to the bowl was 1.27 m.

We then habituated them to side-door removal by placing them individually in the start-box with solid wooden sides in place, to prevent a view of the goal(s). Side-doors were then removed, to allow a view of the conditioned stimuli (feed bowls, placed at the pen entrance which could be viewed through the wire mesh sides of the start-box: Fig. 2). The food bowls were deep enough that at a distance of 1.27 m (diagonal between the start-box and the food bowl) the mealworms could not be viewed. A short test was performed with one hen prior to starting the experiment to ensure that hens were not responding to the sight of mealworms. We found that the hen's behavioural reactions (increased head movements and shorter latencies) were the same towards the spotty bowl whether mealworms were present or absent. Following the viewing period the tunnel-door was lifted to allow access to the goal(s). Hens were considered accustomed to this procedure when they appeared to view the revealed conditioned stimuli during the 10 s viewing period (assessed by observing lateral head movements), and when they entered the tunnel within 10 s of tunnel door removal. In total, approximately 30 unidirectional training trials were carried out per hen. The available goal (empty or mealworm) and its location relative to the start-box (left or right) were systematically varied to ensure equally balanced training for each individual.

### 2.4 Experimental procedure

#### 2.4.1 Experimental conditions

Five experimental conditions were examined:

i. *Unidirectional-Empty* (Uni-E). Unidirectional test. One empty bowl available.
ii. *Unidirectional-Mealworms* (Uni-MW). Unidirectional test. One bowl available, containing mealworms.
iii. *Choice-Empty* (C-E). Choice test. An empty bowl available on each side.
iv. *Choice-Mealworms* (C-MW). Choice test. A bowl containing mealworms available on each side.
v. *Choice-Both* (C-B). Choice test. A bowl containing mealworms available on one side, an empty bowl available on the other side.

All 16 hens were subjected to eight repeats of each experimental condition (total = 640 tests). The order of testing was systematically randomised for each hen, although each session (which comprised five tests) consisted of one test from each experimental condition. Four hens were tested on each experimental day and were given two sessions (one morning and one afternoon) on that day. Each hen was given a total of eight sessions (four morning and four afternoon) across four consecutive weeks. The location of the goal relative to the start-box (left or right) in the unidirectional training trials was systematically allocated, although criteria were set to prevent more than two consecutive tests in the same direction to avoid side-bias development.

#### 2.4.2 Test set-up and protocol

Prior to starting each test, the HR monitor and a stopwatch were activated simultaneously. The test commenced when hens were placed in the start-box and confined for 10 s with the wooden side-doors in place so that a baseline HR could be measured. The side-doors were then removed to reveal the conditioned stimuli and individuals were confined for a further 10 s (the viewing period) before the tunnel-door was raised. When hens reached their goal by entering a pen, the pen-door was closed and they were confined for 120 s to allow the hen to consume mealworms, if available, and for the HR to return to normal. During each test, a maximum time of 300 s was allowed for the hen to leave the start-box. If the hen failed to do so during this time, the test was stopped and the hen was removed from the start-box. If hens left the start-box within the 300 s period, but remained in the tunnel, an additional 120 s was allocated, making the maximum test time 420 s. These times were chosen based on performance during the habituation period.

### 2.5 Measures

#### 2.5.1 Behaviour

Latency to enter a pen was measured for each test. When hens failed to reach the pen in the allotted time (i.e. 300 or 420 s) the maximum time was allocated. To account for potential differences in maximum time, the proportion of allocated time taken to reach the pen was calculated for each test. The number of head movements during the viewing period was recorded using a video camera (SANYO, VCC-6585P, colour CCD camera) which was fixed above the start-box and recordings were captured using a WebCCTV recording system (Quadrox). Data were extracted using The Observer 10.0 software.

#### 2.5.2 HR

The HR was logged onto a micro-SD flash card which was inserted into the HR monitor. The monitor communicated with a base unit (attached to a computer via USB connection) and was controlled using RVC Telemetry Software version 1.5. HR data were extracted using Spike 2 Software (version 6). The average HR (beats per minute) was calculated from two periods: the first 10 s within the start-box (baseline measure), before the hens had any knowledge of which goal(s) would be available; and the 10 s viewing period when the conditioned stimuli were visible.

### 2.6 Statistical analysis

Data were analysed using SPSS Statistics 19 (IBM). All data were normally distributed, with the exception of the data for proportion of allocated time taken to reach the pen, which were right-skewed and therefore log transformed for analysis. A mean measure of HR change (percentage change from baseline to the viewing period), the number of head movements during the viewing period, and the proportion of time taken to reach the pen were calculated from the eight repeats for each hen in each experimental condition. These were then analysed using repeated-measures ANOVAs with experimental condition as the within-subjects factor. *Post hoc* least significant difference (LSD) comparisons were used to examine differences between conditions. Finally, Pearson product moment correlation coefficients were used to analyse the relationship between variables whilst controlling for non-independence due to individual hens.

One hen became distressed during testing and was omitted from the experiment, so analyses considered the outcomes of 600 tests from 15 individuals. When only empty bowls were available, six hens consistently chose to remain in the start-box or tunnel rather than entering a pen (Uni-E = 57/120, C-E = 45/120 trials), but this rarely occurred in any of the conditions including mealworms (2/360 trials). Differences in HR change and head movements were therefore compared for situations when hens did and did not subsequently enter a pen in the conditions including no mealworms. Analyses of these data were performed using MLwiN version 2.25 [27], which allowed multilevel hierarchical models with normalised residuals to be constructed using individual hen and test order as two random levels.

## 3 Results

### 3.1 Change in HR

**Fig. 3:**
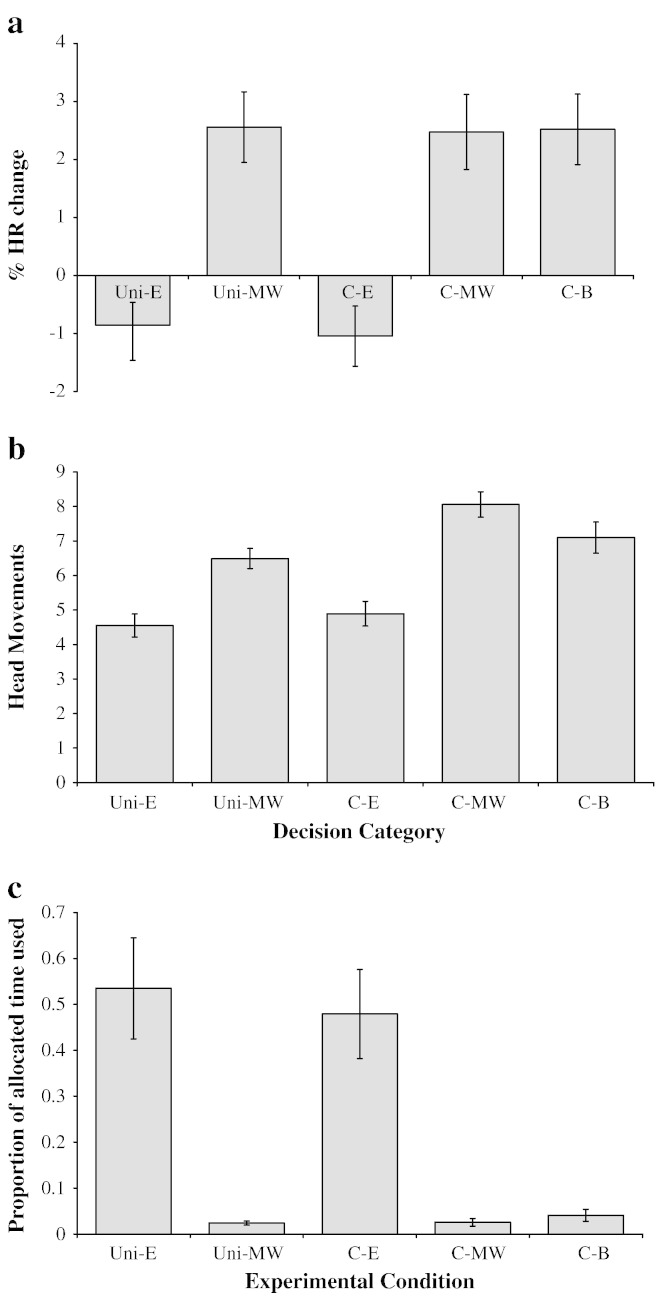
Mean ± 1 SE (a) percentage change in HR from the baseline to the viewing period, (b) number of head movements made during the viewing period, and (c) proportion of maximum allocated time taken to reach the pen for each experimental condition.

The percentage change in HR from baseline to the viewing period was significantly influenced by experimental condition (repeated-measures ANOVA: *F*_4,56_ = 17.03, *p* < 0.001, partial eta squared = 0.549, Fig. 3a). The conditions which offered no mealworms (Uni-E and C-E) were not significantly different to one another (*post hoc* LSD test: *p* = 0.711) and neither were those including mealworms (Uni-MW, C-MW and C-B; all *p* > 0.870). However, the HR change from baseline to the viewing period in Uni-E and C-E was significantly lower than in Uni-MW, C-MW and C-B (all *p* < 0.001).

### 3.2 Number of head movements

There was a significant effect of experimental condition on the number of head movements during the viewing period (repeated-measures ANOVA: *F*_4,56_ = 63.01, *p* < 0.001, partial eta squared = 0.818, Fig. 3b). There was no significant difference between the conditions which included no mealworms (Uni-E and C-E; *post hoc* LSD test: *p* = 0.094), but significantly fewer head movements were measured in these categories than in all conditions including mealworms (Uni-MW, C-MW and C-B; all *p* < 0.001). In condition C-B there tended to be more head movements made than in Uni-MW (*p* = 0.053), and significantly more head movements were seen in C-MW compared with all other categories (all *p* < 0.012).

### 3.3 Proportion of allocated time to reach the pen

The proportion of time taken to reach the pen was significantly influenced by condition (repeated-measures ANOVA with a Greenhouse–Geisser correction for sphericity: *F*_2,33_ = 52.70, *p* < 0.001, partial eta squared = 0.790, Fig. 3c). Hens took a significantly greater proportion of time to reach the pen in the conditions with no mealworms (Uni-E and C-E) compared with the categories including mealworms (Uni-MW, C-MW and C-B; *post hoc* LSD test: all *p* < 0.001). There were no significant differences between Uni-MW, C-MW, C-B (all *p* > 0.513) or between Uni-E and C-E (*p* = 0.919). Only five hens entered a pen in all eight repeats in all experimental conditions, but qualitatively the same results were obtained when considering only these latencies.

### 3.4 Correlation between measures

The percentage change in HR from the baseline to the viewing period, the number of head movements during the viewing period and the proportion of allocated time to reach the pen were found to be significantly correlated when controlling for individual hen (Pearson product moment correlation coefficient: all *p* < 0.001). HR change and head movements were positively correlated (correlation coefficient = 0.238), whereas the time taken to reach the pen was negatively correlated with both HR change (correlation coefficient = − 0.351) and head movements (correlation coefficient = − 0.496).

### 3.5 Association between arousal during the viewing period and subsequent stay/enter choice

The following model was constructed to examine the effect of condition (Uni-E or C-E) and subsequent choice (entered pen or not) on the number of head movements during the viewing period, with individual hen (*i*) and test order (*j*) as two separate random levels:

$$\mathrm{Head}\;\mathrm{movement}s_{\mathrm{ij}}=4.020{\left(0.335\right)}_{0\mathrm{ij}}+1.000{\left(0.335\right)}_{\mathrm{ENTERED}\;\mathrm{PEN}\mathrm{ij}}+0.251{\left(0.219\right)}_{C‐E\mathrm{ij}}.$$

**Fig. 4:**
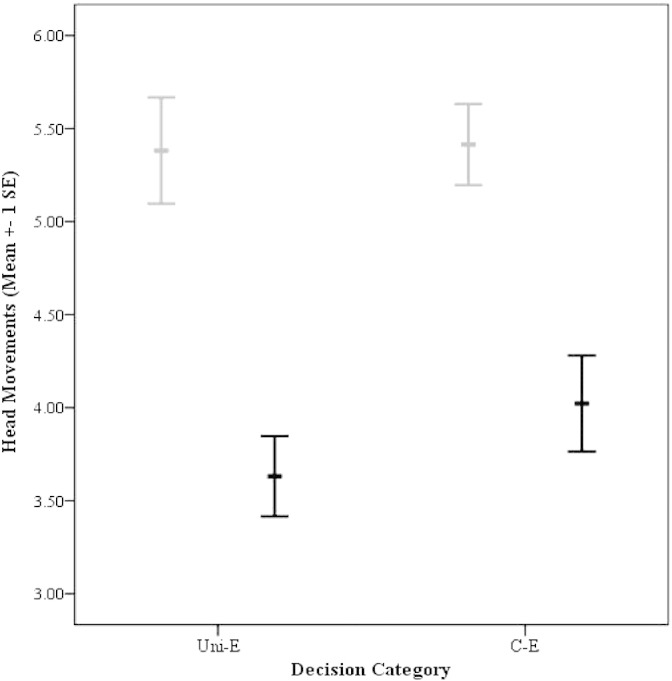
Mean ± 1 SE number of head movements made during the viewing period for experimental conditions Uni-E and C-E when hens ultimately did (in grey) and did not (in black) enter a pen.

Five hens consistently entered a pen when the only available goal was the empty bowl, whereas the other 10 were less consistent and would often remain in the start-box or tunnel for the entire test. Overall, hens made significantly more head movements during the viewing period preceding occasions that they entered a pen (*n* = 138/240) compared to when they remained in the start-box or the tunnel (*n* = 102/240) (coefficient = 1.000, s.e. = 0.335, *n* = 240, *p* = 0.003, Fig. 4). These results were unaffected by experimental condition (coefficient = 0.251, s.e. = 0.219, *n* = 240, *p* = 0.252), although in condition C-E hens tended to enter a pen more often than in condition Uni-E (paired samples t-test: *t*_14_ = 1.87, *p* = 0.082).

The following model was constructed to examine the effect of condition (Uni-E or C-E) and subsequent choice (entered pen or not) on the HR change between the baseline and viewing period, with individual hen (*i*) and test order (*j*) as two separate random levels:

$$\mathrm{HR}\;\mathrm{Chang}e_{\mathrm{ij}}=-1.163{\left(0.596\right)}_{0\mathrm{ij}}+0.641{\left(0.657\right)}_{\mathrm{ENTERED}\;\mathrm{PEN}\mathrm{ij}}+-0.285{\left(0.473\right)}_{C‐E\mathrm{ij}}.$$

HR change between the baseline and the viewing period did not differ significantly between situations where hens entered a pen or when they stayed in the start-box or tunnel (coefficient = 0.641, s.e. = 0.657, *n* = 240, *p* = 0.329). These results were also unaffected by experimental condition (coefficient = − 0.285, s.e. = 0.473, *n* = 240, *p* = 0.547).

## 4 Discussion

We found similar patterns of response in both HR and head movements to the detection and anticipation of a mealworm reward (conditions Uni-MW, C-MW and C-B). An increase in HR between the baseline and viewing period was observed in these conditions, whereas a decrease in HR occurred when no mealworms were available (Uni-E and C-E). Significantly more head movements were also found during the viewing period when a mealworm reward was available, which fits with previous work investigating behavioural indicators of arousal in anticipation of a mealworm reward in chickens [18]. Shorter latencies to reach the pen when mealworms were present confirm that hens were more motivated to obtain this reward [26]. The three measures were also correlated and, combined, suggest that hens experienced increased arousal in anticipation of a high-quality goal and, crucially, that these measures were detectable around the time of decision-making. Thus, both our physiological and behavioural measures seemed to detect arousal associated with previous experience of the available options, as required by the somatic marker hypothesis [8,9,11].

The somatic marker hypothesis requires not only the elicitation of a simple conditioned response to a reward, but also that arousal plays some role in the decision-making [8]. Although some of our results obtained during the viewing period could be due to a conditioned response, we were careful to ensure that hens were not responding to a direct perception of mealworms, by making sure that they were not visible from the start-box. Other factors that are integral to decision-making include having more than one goal available, having different goals available enabling a choice or having different response options available. If the somatic marker hypothesis is relevant in non-human animals, we would expect to see different patterns of arousal in our experimental conditions that manipulated these aspects.

When comparing the number of goals available (i.e. one vs. two), significantly more head movements were observed when hens were presented with two mealworm bowls (C-MW) than in any other condition, suggesting that hens were in fact more aroused in this test condition [18]. However, this effect was not observed when comparing responses to one (Uni-E) and two (C-E) empty bowls, which could indicate that empty bowls were not sufficiently arousing in general. Interestingly, we found no significant differences in the HR change from baseline to the viewing period when comparing the perception of one or two goals, whether mealworms or an empty bowl. It is possible that a physiological maximum HR was reached in response to the perception of one mealworm goal, so no difference in this measure was observed when two goals were perceived. These results suggest that the perception of an increased number of available appetitive goals increased behavioural arousal, which may reflect the difference in the complexity of a decision involving single and multiple available goals. In a human gambling task, subjects showed increased arousal when given a choice of bet size (active choice) compared with when they were given a fixed amount to gamble with (no-choice) [28], suggesting that active choice intensifies the role of emotional arousal during risky decision-making. The present study is the first, to our knowledge, to examine arousal responses in non-human animals to the number of available goals.

The test condition in which chickens were presented with a choice between the goals of greater and lesser value (C-B) provided the only situation in which the outcome was uncertain. That is, it was possible for hens to make a “mistake” (by choosing the empty bowl over the mealworms), and thus this treatment may have been perceived as a more “risky” decision [29,30]. One fundamental precept of the somatic marker hypothesis is that emotions help to guide decisions, particularly in uncertain or “risky” situations [31]. As such, it might have been expected that arousal would be highest during the viewing period in category C-B, but we found no evidence of this. In this condition, hens chose mealworms most of the time (96%), showing a clear preference for them over the empty bowl, as was found in previous work [25]. It is therefore possible that this condition represented a fairly “easy”, low-risk decision. In human gambling tasks, exaggerated anticipatory skin conductance responses were generated only when decisions were perceived as “risky” [32]. Further work should therefore be conducted to investigate whether arousal in non-humans is affected by decision risk in other contexts, or when decisions may be perceived as more “difficult” (e.g. if reward size is balanced by the difficulty of reward access).

The other type of decision available to hens in this experiment was whether or not to leave the start-box or tunnel and enter a pen at all. There was considerable variation in this decision when no mealworms were available. There were no significant differences in HR during the viewing period on occasions when hens subsequently chose to enter a pen and on occasions when they chose not to but, again, a different pattern of response was noted for the head movements. In this case, the number of head movements made during the viewing period strongly predicted whether a hen would subsequently enter a pen containing an empty bowl, with an average of 30% more head movements made prior to entering a pen than when they remained in the T-maze. This result suggests, as has been proposed by human psychologists [10–12,33], that arousal during decision-making may be related to decision outcome, providing preliminary support for the somatic marker hypothesis. Generally, our results suggest that head movements may provide a more sensitive measure of arousal than HR during decision-making in chickens.

In summary, we have for the first time successfully monitored arousal around the time of decision-making in a non-human model, whilst comparing responses to the number of available goals. Both HR and head movements increased during the viewing period, in anticipation of a high-value reward. In contrast, only head movements discriminated between conditions where the number of available goals was varied and were associated with simple stay/enter decisions. The physiological and, especially, the behavioural results presented here provide an important foundation for investigating the role of arousal during more complex or difficult animal decisions. Future work should examine whether differential individual patterns of arousal are associated with making better or worse decisions in more complex or difficult tasks, a fundamental requirement of the somatic marker hypothesis.
